# Role of intermediate water in alleviating postsurgical intrapericardial adhesion

**DOI:** 10.1007/s00595-024-02953-4

**Published:** 2024-11-08

**Authors:** Tatsuya Suzuki, Hayato Konishi, Akiyo Suzuki, Takahiro Katsumata, Yasuhiro Fukuda, Koki Miyamoto, Tomokazu Ise, Yukiko Tanaka, Aki Yamamoto, Panyue Wen, Shohei Shiomoto, Masaru Tanaka, Shintaro Nemoto

**Affiliations:** 1 Department of Thoracic and Cardiovascular Surgery, Osaka Medical and Pharmaceutical University, 2-7 Daigaku-Machi, Takatsuki, Osaka 569-0801 Japan; 2 Research and Development Center, The Japan Wool Textile Co., Ltd, Kakogawa, 440 Funamoto, Yoneda-cho, Hyogo 675-0053 Japan; 3 Soft Materials Chemistry, Institute for Materials Chemistry and Engineering, Kyushu University, Fukuoka, 744 Motooka, Nishi-ku, Fukuoka 819-0395 Japan

**Keywords:** Cardiac surgery, Postoperative pericardial adhesion, Antiadhesive, Polymer, Intermediate water

## Abstract

**Purpose:**

Various polymers have been used as postsurgical antiadhesive materials; however, the mechanisms underlying their efficacy remain unclear. Intermediate water has been found to prevent the adhesion between polymer molecules and proteins or cells. The present study investigated the role of intermediate water retained in the polymer in alleviating postsurgical pericardial adhesion.

**Methods:**

Hydrophobic fabrics were prepared using biodegradable polyglycolic acid. To add intermediate water, the fabric fibers were coated with poly(oxyethylene)oleyl ethers. Intermediate water in the hydrated state was detected by a thermal analysis for each material, and cell attachment to the fibers with or without coating was observed in vitro. Using a canine model of postsurgical pericardial adhesion, the severity of adhesion was examined along with a histological assessment during treatment, with or without fabric coating.

**Results:**

Intermediate water was detected in the coating materials but not in polyglycolic acid. Coating significantly reduced the cell attachment to the fibers. Coating also alleviated adhesion by reducing inflammation in the fibrous layer and replacing the fabric and granulomas that develop around the surgical sutures in the pericardial space.

**Conclusions:**

Intermediate water in the hydrated polymer of anti-adhesives may play an important role in alleviating postoperative pericardial adhesion.

## Introduction

Postoperative intrapericardial adhesion is an inevitable complication that often makes repeat surgery challenging due to bleeding, tissue damage, and long operation times caused by the need for adhesion dissection. Intrapericardial adhesion has also been recognized as a tissue healing mechanism for mesothelial damage [1]. The process is initiated by the attachment of fibrin bundles within 1 week after surgery, followed by an inflammatory reaction with macrophages, fibroblasts, and microangiogenesis within 1 month. After the subsidence of inflammation, fibrous attachments with mesothelial recovery develop as adhesions 1 month after surgery. The degree of adhesion varies depending on the surgical invasiveness, bleeding, and cardiopulmonary bypass.

Various materials are used to prevent adhesion, such as a physical barrier between the epicardium and pericardium. Naturally derived polymers such as oxidized regenerated cellulose [2], hyaluronic acid [3], dextrin [4], and gelatin [5] have been successfully applied clinically. These hydrophilic materials transform into hydrogels and degrade within 1 month in vivo, leaving only modest adhesion.

These naturally derived polymers contain a substance known as “intermediate water” [6–9]. Intermediate water is loosely bound to the hydrated polymer and inhibits the adhesion of platelets and fibrinogen to the polymer [10–12]. We propose the “intermediate water concept,” which hypothesizes that the presence of intermediate water is essential for improving biocompatibility of materials. This property may allow polymers to inhibit fibrin and cell attachment to the surface of the epicardium and pericardium during the critical phase of adhesion.

The present study investigated the role of intermediate water in alleviating postoperative intrapericardial adhesion.

## Materials and methods

### Ethics statement

This study was approved by the Institutional Animal Care and Use Committee of the Osaka Medical and Pharmaceutical University (approval ID #21,069-A). All animals received humane care according to the guidelines of the committee and the National Institutes of Health Guidelines for the Care and Use of Laboratory Animals.

#### *Hypothesis*

The hypothesis of this study was that a hydrophobic polymer without intermediate water could alleviate intrapericardial adhesion when coated with a hydrophilic polymer containing intermediate water. Polyglycolic acid (PGA) was selected as a hydrophobic aliphatic polymer. The clinically used PGA sheet is known to have no anti-adhesion effect but induces fibrous tissue formation and is thus used for tissue repair and reinforcement [13, 14]. poly(oxyethylene)oleyl ether (PO-OE), a nonionic surfactant for smooth knitting known to enhance drug absorption [15], was chosen as the hydrophilic polymer for coating PGA. Two PO-OEs (PO-OE 66–1 and PO-OE 98–2, FL-IN1; Inuisyoji Corp., Osaka, Japan) were blended to maintain an ideal hydrophile–lipophile balance, which is the standard for ensuring proper hydrophilicity in the coating materials.

In this study, we investigated whether or not the PO-OE coating could cancel the aforementioned properties of the PGA sheet and provide an antiadhesion effect.

### Preparation of a sheet of PGA and coating with PO-OE

**Fig. 1:**
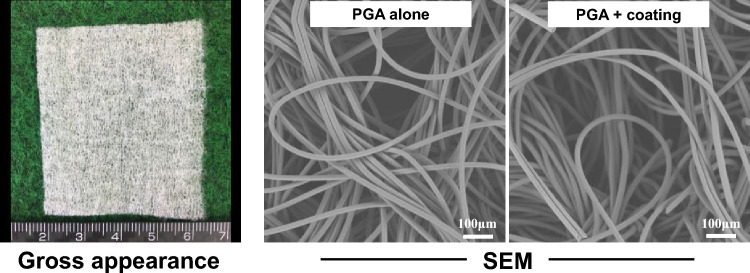
Gross appearance and scanning electron microscopy (SEM) images of polyglycolic acid (PGA) sheets. Coating material: a mixture of two types of poly(oxyethylene)oleyl ether

The PGA sheet was manufactured as circular knitted fabric composed of PGA yarn. The yarn consisted of ten filaments with an individual filament diameter of 22.2 μm. An aqueous emulsion of the mixed PO-OEs was used to coat multifilament yarns by dipping spun PGA fibers (Pellets; BioDegmer, BMG Inc., Kyoto, Japan). This process is required to transform the multifilament yarn into a bundle. The fabric was needle-punched to control stretchability and dimensional stability. The weight and thickness of the sheet were 70–80 g/m^2^ and 0.66 mm, respectively. To obtain non-coated PGA sheets, the coated PGA sheets were ultrasonically washed with 80% ethanol to remove PO-OE. Finally, the sheets were packaged and sterilized using ethylene oxide gas. The sheets were then stored at room temperature for a further evaluation. Gross and scanning electron microscopy images of the sheets are shown in Fig. 1.

### In vitro degradation of PGA sheets with and without PO-OE coating

A 50 mg PGA sheet with or without PO-OE coating was immersed in 40 mL of 1/15 mol/L phosphate-buffered saline (PBS). The immersion periods were 0, 1, 2, 3, and 4 weeks, and the PGA sheets were maintained in an incubator at 37 °C. After immersion, the PGA sheets were removed, washed with distilled water, and vacuum-dried (*N* = 5 each with and without coating). To evaluate the extent of PGA degradation, the weight-averaged molecular weight (MW) of each polymer was analyzed by gel permeation chromatography using a previously reported method [16]. In brief, polymers were extracted from excised PGA samples using hexafluoro-2-propanol (HFIP), and the tissue residue was removed by filtration through deactivated glass wool. Measurements were performed using gel permeation chromatography (Prominence GPC; Shimadzu Corp., Kyoto, Japan) with a refractive index detector for PGA. The MW of PGA was calculated from a calibration curve obtained using polystyrene standards (MW range 0.5–2110 kDa).

### Differential scanning calorimetry

To detect intermediate water in the materials, differential scanning calorimetry (DSC) measurements of hydrated samples were performed as described in previous studies [17, 18]. In a DSC analysis, hydration water can be identified as free water (very weakly bound water), intermediate water (loosely bound water/freezing bound water), and non-freezing water (tightly bound water) by cold crystallization followed by a heat-melting process [17–20]. Intermediate water is cold-crystallized in a range of −40 to −50 °C and melts below 0 °C, at which point free water melts. In brief, pieces of the PGA sheets were immersed in pure water. The sample sheet (4.3 mg) was sealed in an aluminum cell (GCA-0052; Hitachi High-Tech Science Corp., Tokyo, Japan). The samples were cooled to −100 °C at a rate of 5 °C/min, held for 5 min, and then heated to 50 °C at the same rate using the DSC equipment (DSC7000X; Hitachi High-Tech Science Corp.). Viscous liquid PO-OEs were mixed with water at 50 °C for 5 days to obtain a homogenous mixture, which was then used for DSC measurements under the same conditions. The mass of the PO-OE 66–1/water system was 3.4 mg, and that of the PO-OE 98–2/water system was 4.7 mg. The water content in each system was 2 g/g. Intermediate water is defined as hydrated water that exhibits crystallization or melting at temperatures lower than the phase-transition temperature of pure water [20, 21].

### Cell attachment to PGA sheets with and without a PO-OE coating

Tissue culture 24-well plates composed of polystyrene were pretreated with 0.5% (w/v) poly(n-butyl methacrylate 70-co-2-methacryloyloxyethyl phosphorylcholine 30) and left to dry overnight. Three sheets (*N* = 3) of each PGA sample were placed on plates and sterilized using UV light for 1 h. Subsequently, the wells were thoroughly rinsed with PBS to eliminate any contaminants. A cell culture solution (DMEM/F12) containing 10% fetal bovine serum (FBS) was added to the wells and incubated for 1 h at 37 °C to promote cell attachment. Normal human dermal fibroblasts (NHDFs) were then seeded on the samples at a density of 1 × 10^4^ cells/cm^2^ and allowed to adhere to and proliferate on the surface of the samples for 1 h and 1, 3, and 7 days. The culture medium was replenished every 3 days to maintain optimal cell growth conditions. After removal of the medium, the cells were treated with 1000 μL of fresh medium and 100 μL of Cell Counting Kit-8 solution (343–07623; Dojindo, Kyoto, Japan). After 2 h incubation at 37 °C, the culture medium was subjected to a spectrophotometric analysis at 450 nm using a microplate reader (Infinite 200PRO M Plex; Tecan, Zürich, Switzerland). After staining with crystal violet solution to visualize the cells, images were captured using a fluorescence microscope (BZ-X710; Keyence, Osaka, Japan).

### Induction of intrapericardial adhesion and application of a PGA sheet on the surface of the heart

**Fig. 2:**
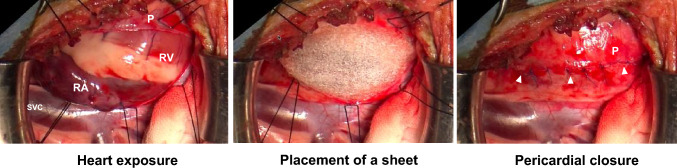
Induction of intrapericardial adhesion and application of a polyglycolic acid sheet on the surface of the heart using a pericardial incision (approximate position shown by white arrowheads). P: pericardium, RA: right atrium, RV: right ventricle, SVC: superior vena cava

Twelve adult male beagles (age 14.4 ± 3.5 months, body weight 10.6 ± 0.9 kg; Oriental Bioservice Inc., Kyoto, Japan) were used in the study. Surgery to place a sheet on the surface of the heart was performed with reference to a previously reported technique [22], with some modifications. In brief, the animals were intubated after induction with an intravenous injection of thiamylal sodium (25 mg/kg), and general anesthesia was maintained with inhaled sevoflurane. The right chest was opened by standard thoracotomy, and the heart was exposed by gentle retraction of the lungs. An approximately 10-cm longitudinal incision was made in the anterior pericardium parallel to the phrenic nerve (Fig. 2). The epicardial surfaces of the right atrium (RA) and right ventricle (RV) were damaged using a rasping file for electrocautery. Blood (5 mL) was collected through a purse-string suture on the RA wall using a 5–0 polypropylene suture (Prolene; Ethicon, Cincinnati, OH, USA) [22, 23]. Four additional single stitches were placed on the RA and RV using the same nonabsorbable suture to induce granulation. Blood was sprinkled into the inter-pericardial space. After placing a rectangular sheet over the RA and RV (Fig. 2), the pericardial incision was directly approximated using multiple single stitches with the same non-absorbable suture (Fig. 2). The chest was then closed. An intramuscular injection of butorphanol tartrate (0.2 mg/kg) was administered for analgesia before the animals awakened. Meloxicam (2.5 mg) was administered orally for analgesia 3 days after surgery. All animals were housed until a further assessment, as described below.

### Gross assessments of intrapericardial adhesion and tissue sample harvesting

Three or six months after sheet implantation (two animals in each period and three groups described below), the animals were anesthetized as described above, and repeat thoracotomy was performed through the previous incision. After careful dissection of the lung adhesions, the entire outer surface of the pericardium was exposed. The pericardial space was then entered, and pericardial adhesions were dissected. The dissection procedure was video-recorded. A macroscopic evaluation was performed on-site and later by two surgeons using a previously reported scoring system [24] with some modifications (Table 1). After dissection, the animals were humanely euthanized via intravenous administration of a fatal dose of thiamylal sodium (100 mg/kg), in addition to inhaled sevoflurane. A sample block was harvested via a longitudinal incision across the RA to the right ventricle (RV), including the area to which the sheet was applied. The samples were collected for histological analyses.

**Table 1:** Definition of the adhesion score

Score	Definition
0	No adhesions: no surgical procedure required
1	Mild adhesion: dissectible by simple traction
2	Moderate adhesion: dissectible with scissors without tissue damage
3	Tight adhesion: requires an electrocautery, occasionally with tissue damage
4	Strong adhesion: not dissectible

### Histological assessments of intrapericardial adhesion

The excised tissue blocks were fixed in 10% formaldehyde. A sample slice was cut longitudinally and embedded in paraffin. Slices were prepared using microtome sectioning and stained with Masson’s trichrome (MT). The thickness of the pericardial space at the site of PGA sheet implantation or at the equivalent site was measured at five arbitrary sites in each animal. Immunostaining was performed to observe inflammatory reactions by targeting macrophage-specific ionized calcium-binding adapter molecule 1 (Iba1; anti-Iba1 antibody-GTX100042; Gene Tex Inc., Irvine, CA, USA). Iba1 cells were visualized with 3;3’-diaminobenzidine tetrahydrochloride (DAB kit 10-0048RUO; Sakura Fintek USA, Torrance, CA, USA). Inflammatory and fibrous reactions in the pericardial space and the surface of the epicardium and pericardium and morphological changes in the sheet were evaluated microscopically.

### Grouping of animals to compare intrapericardial adhesion between different sheets

To assess the efficacy of intermediate water with or without coating material (PO-OE) in reducing the severity of intrapericardial adhesion, the animals were divided into three groups according to the sheet applied on the surface of the heart: Group N, control without any sheet; Group Y, PGA sheet without coating; and Group Y + C, PGA sheet with PO-OE coating.

### Statistical analyses

The Wilcoxon/Kruskal–Wallis test was used to compare data between two groups for changes in molecular weight with and without immersion in PBS and the cellular behavior of NHDF adhesion with and without coating on the PGA sheets. The mean values from a one-way analysis were compared using Student’s *t* test for each of the two groups.

A value of *p* < 0.05 was used to indicate a significant difference in all analyses. All calculations were performed using the JMP Pro 16 software program (SAS Institute Inc., Cary, NC, USA).

## Results

In vitro *degradation of PGA sheets with and without PO-OE coating.*

**Fig. 3:**
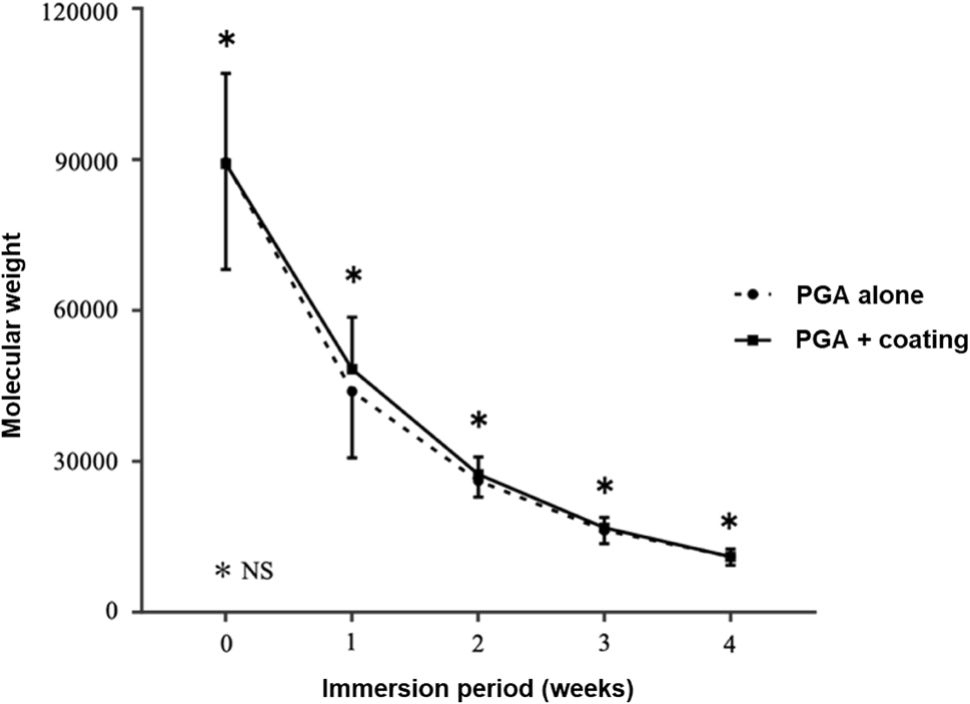
In vitro degradation after immersion in phosphate-buffered saline of polyglycolic acid (PGA) sheets with and without coating with poly(oxyethylene)oleyl ether. NS: not significant

The molecular weights of PGA sheets with and without PO-OE coating gradually decreased upon immersion in PBS, with no significant difference observed over time (Fig. 3).

### Water states of hydrated PGA and PO-OE

**Fig. 4:**
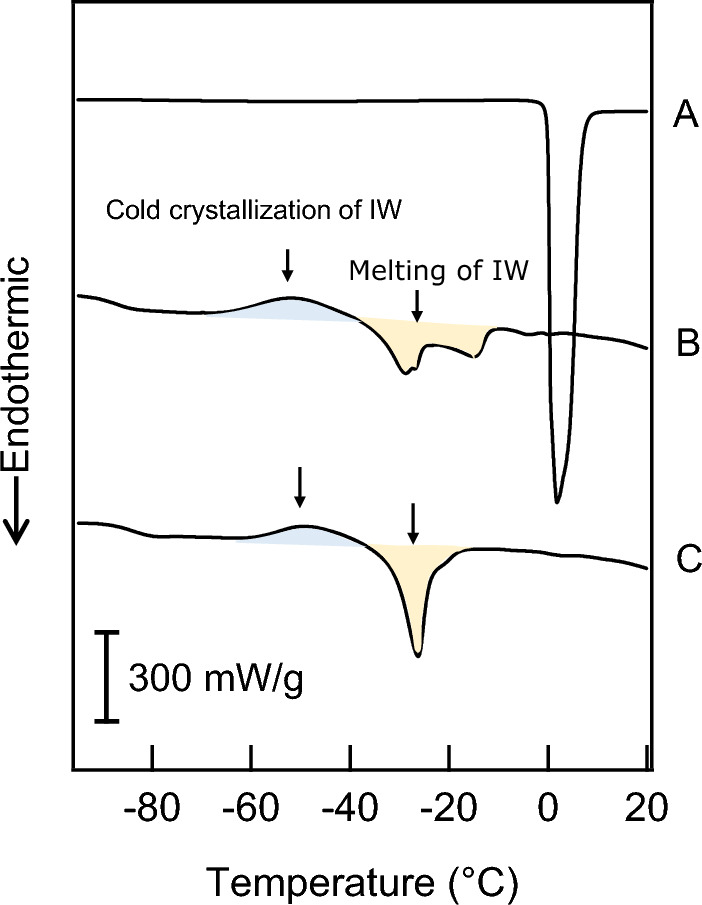
Differential scanning calorimetry curves of hydrated samples recorded during heating. **A** Polyglycolic acid. **B** Poly(oxyethylene)oleyl ether (PO-OE) 66–1. **C** PO-OE 98–2. IW: intermediate water

The hydrated samples exhibited different DSC curves (Fig. 4). The two PO-OEs exhibited exothermic peaks at approximately −50 °C during the heating process, indicating cold crystallization of the intermediate water. Endothermic peaks were observed at approximately −30 °C, indicating melting of the intermediate water. The DSC results showed that the PO-OEs were able to bind to the intermediate water. In contrast, hydrated PGA showed no peaks, except at 0 °C, indicating the presence of free water.

### Cell attachment to PGA sheets with and without PO-OE coating

**Fig. 5:**
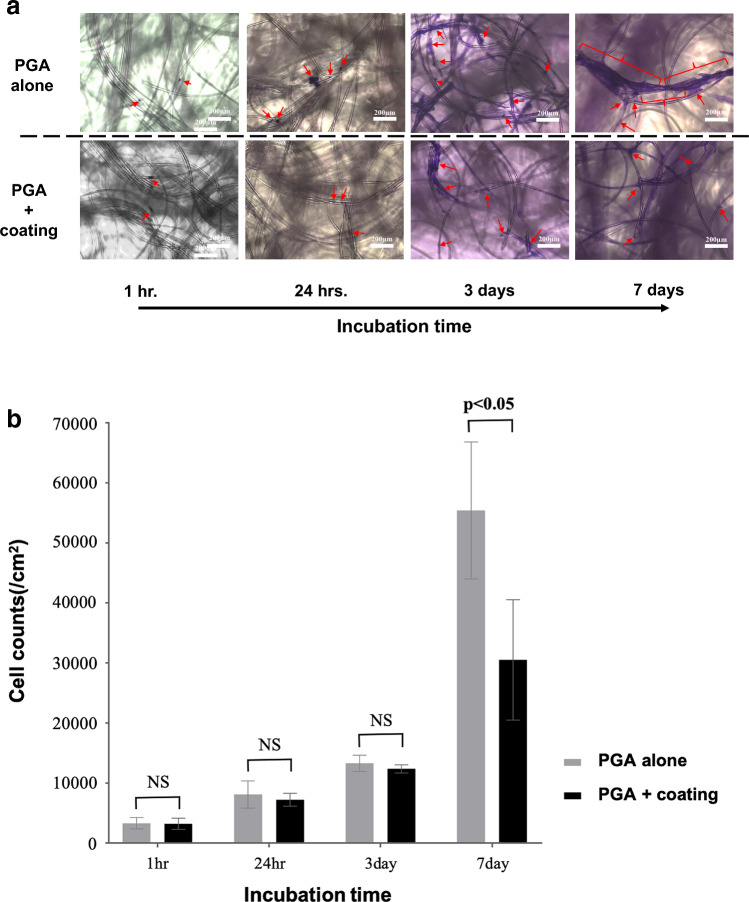
**a** Representative photographs of attached or proliferated fibroblasts detected by crystal violet staining of fibers of polyglycolic acid (PGA) sheets with or without coating with poly(oxyethylene)oleyl ether. Red arrows: location of fibroblasts; red curly brackets: cell clusters. **b** Cell counts of fibroblasts attached to the fibers of PGA sheets with and without coating. *P* < 0.05 by the Wilcoxon/Kruskal–Wallis test. NS: not significant

Representative photographs clearly show that the PO-OE coating inhibited NHDF adhesion to PGA fibers (Fig. 5a). Quantitative cell counts of NHDFs attached to the fibers confirmed the significant inhibitory effects of the PO-OE coating on fiber proliferation after 7 days of incubation (Fig. 5b).

### Gross assessment of intrapericardial adhesion during dissection (Fig. 6)

**Fig. 6:**
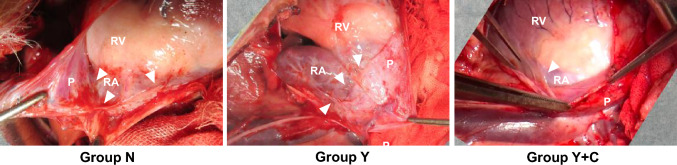
Representative gross appearance of intrapericardial adhesion (white arrowheads) at 6 months after surgery. Group N: no sheet; Group Y: PGA sheet without coating; Group Y + C: PGA sheet with coating. P: pericardium; RA: right atrium; RV: right ventricle

Adhesion scores for each animal are listed in Table 2. In Group N, at both 3 and 6 months after surgery, the pericardial surface and epicardium directly adhered to the site of surgical manipulation to dissect adhesion. Therefore, hemostasis is often required during adhesion dissection. The adhesion was entirely released 3 months after surgery, but the epicardial surface was damaged with bleeding due to firm adhesion at 6 months, especially at the sutures placed on the RA. In Group Y, mild intrapericardial adhesion with a thin fibrous layer was present between the pericardial surface and epicardium at both timepoints, but the adhesion was completely dissected out in the fibrous layer more easily at 6 months than at 3 months. In Group Y + C, the findings for adhesion were similar to those in Group Y. However, dissection of the adhesion was accomplished with greater ease in Group Y + C than in Group Y at both timepoints.

**Table 2:** Adhesion scores in each animal

Group	N		Y		Y + C	
Antiadhesive	None		PGA		PGA + coating	
Postoperative period (months)	3	6	3	6	3	6
Right atrium	3, 2/2, 2	3, 3/3, 2	3, 2/3, 2	1, 2/1, 2	3, 2/2, 2	2, 2/1, 2
Right ventricle	0, 0/0, 0	0, 2/0, 1	0, 2/0, 0	0, 0 /0, 1	0, 0/0, 1	0, 0/0, 0
Sutures on right atrium	3, 1/3, 1	4, 3/2, 3	3, 2/3, 3	1, 2/2, 2	2, 2/2, 2	2, 2/2, 2
Pericardiotomy closure line	3, 2 /3, 2	2, 2/3, 2	3, 2/2, 2	1, 2/2, 2	2, 2/2, 2	2, 2/2, 2

Each column shows scores for two animals evaluated by two independent surgeons

*PGA* polyglycolic acid

### Histological assessments of intrapericardial adhesion

**Fig. 7:**
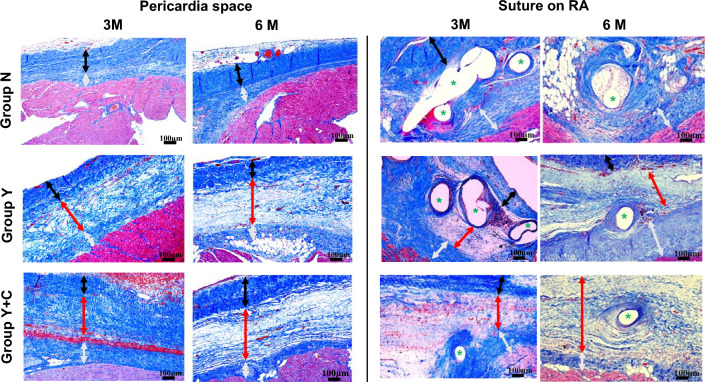
Representative microscopic images of the intrapericardial space and fibrous tissue around the sutures placed on the right atrium on Masson’s trichrome staining at 3 and 6 months after surgery. Group N: no coating; Group Y: PGA sheet without coating; Group Y + C: PGA sheet with coating. Scale bar = 100 μm. Arrows: black, pericardium; gray, epicardium; red, intrapericardial space. *Non-absorbable sutures

**Fig. 8:**
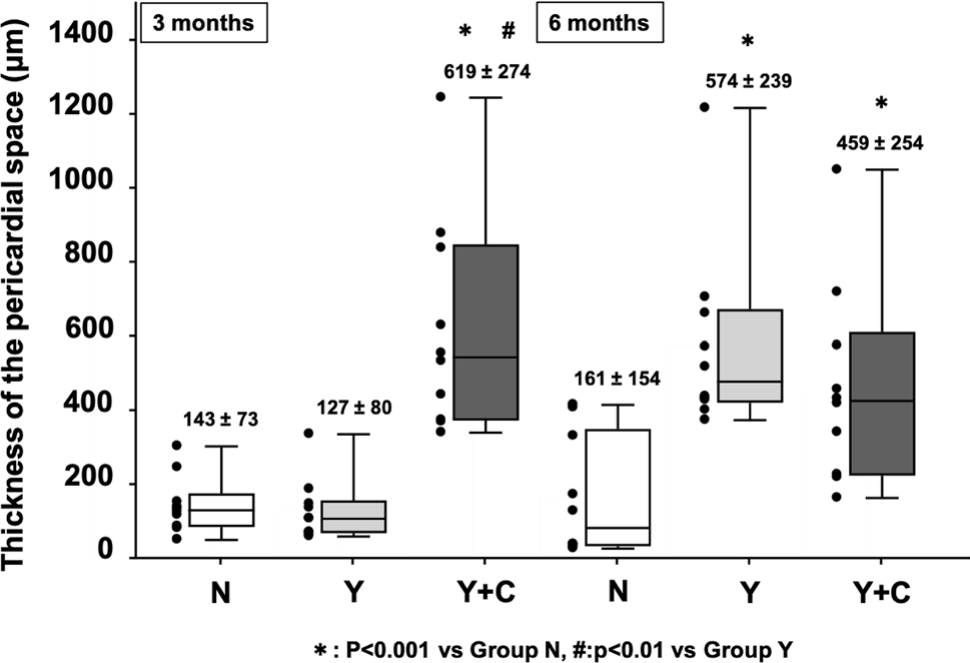
Scatter and box plots of the thickness of the pericardial space at 3 and 6 months after surgery in a canine pericardial adhesion model. Group N: no coating; Group Y: PGA sheet without coating; Group Y + C: PGA sheet with coating

In Group N, although the mesothelial cells were well preserved over the surface of the pericardium and epicardium, the pericardium and epicardium were directly attached at both 3 and 6 months after surgery (Fig. 7, pericardial space). Interestingly, cell infiltration and fibrous tissue growth were not observed after 3 months. However, collagen fibers proliferated slightly on the surface of the pericardium at 6 months postoperatively. In Groups Y and Y + C, the PGA sheets were completely absorbed and replaced by a connective tissue layer in the pericardial space at each postoperative timepoint. In Group Y, the connective tissue layer irregularly eroded the pericardium and epicardium 3 months after surgery, whereas this active reaction was modest in Group Y + C at this timepoint. The border of the connective tissue layer became clear with sparse collagen fibers and less cell infiltration in Groups Y and Y + C. Three months after surgery, suture-reactive granulomas had formed in all groups (Fig. 7, suture on RA). Granuloma was markedly developed in Groups N and Y (Fig. 7), whereas the reaction was modest in Group Y + C. Although the granuloma was replaced with a fibrous scar 6 months after surgery in all groups, the scar in Group N was prominent with collagen infiltration into the myocardium, resulting in tight fibrotic adhesion. In contrast, fibrosis around the suture was sparse, with a clear border in Group Y + C at 6 months after surgery. At 3 months after sheet insertion, the pericardial space was significantly thicker in Group Y + C than in groups N and Y, although the thickness did not significantly differ between them (Fig. 8). In contrast, the thick pericardial space of Group Y + C became similar to that of Group Y after another 3 months and was significantly thicker than that of Group N (Fig. 8).

**Fig. 9:**
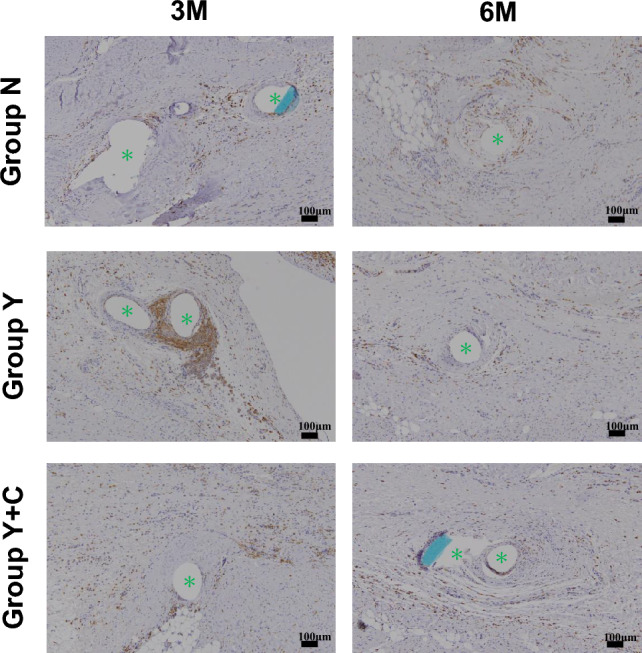
Representative microscopic images of immune-stained macrophages infiltrating near the surgical suture in the pericardial space at 3 and 6 months postoperatively. Group N: no coating; Group Y: PGA sheet without coating; Group Y + C: PGA sheet with coating. Scale bar = 100 μm. Brown spots indicate the visualized macrophages. *Non-absorbable sutures

Locating macrophages via Iba1 immunostaining showed that Iba1-positive cells strongly aggregated against the sutures and spread into the pericardial space in Group Y just 3 months after implantation (Fig. 9). In the other groups and during the postoperative observation period, Iba1-positive cells were only slightly scattered in the pericardial space (Fig. 9).

This pathology reflected the gross findings of difficulty in dissecting the adhesions.

## Discussion

This study had three major findings. First, PO-OEs contain intermediate water in their hydrated state, and coating with PO-OEs can provide intermediate water to PGA fibers, which cannot have intermediate water. Second, in the in vitro study, PO-OE coating inhibited the attachment and proliferation of fibroblasts onto PGA fibers. Third, although it was not possible to prevent the undesirable replacement of the PGA sheet by fibrotic tissue in the pericardial space in vivo, the addition of coated PO-OEs alleviated the inflammatory response induced by PGA 3 months after implantation. This was thought to be reflected in the formation of sparse connective tissue during adhesion-release surgery.

An ideal antiadhesion material has a shielding function and prevents adverse inflammatory responses that initiate fibrous proliferation and cell attachment to the epicardium and pericardium, resulting in adhesion. To select a polymer, it is necessary to re-evaluate the mechanism of cell attachment. Recently, it was found that intermediate water, loosely bound to specific polymers when hydrated, plays an important role in biocompatibility by inhibiting the adhesion of proteins and cells to the polymer [20, 25, 26]. Polymers containing intermediate water, such as poly(2-methoxyethyl acrylate) (PMEA), have been applied in clinical practice as biocompatible coating materials for extracorporeal circuit tubes and oxygenators to prevent thrombosis by inhibiting protein and platelet attachment to the material surface [27]. At present, the main polymers used as commercially available antiadhesives are naturally derived polymers [2–5]. These polymers transform into hydrogels in vivo and exert excellent antiadhesive effects. Interestingly, intermediate water molecules have been detected in these polymers [6–9].

To address the role of intermediate water in preventing intrapericardial adhesion, we examined the placement of polymers with or without intermediate water in the pericardial space. Because it is impossible to remove the intermediate water from the hydrogels, we chose an alternative method of adding intermediate water to PGA, a biodegradable polymer used in clinical practice. Woven fabric PGA, which has been used clinically to prevent air leakage in lung resection surgery, induces nonspecific inflammatory reactions around PGA fibers during the first two weeks and is replaced by fibrous tissue adhering to the pleural surface after eight weeks in the pleural space [14]. We examined whether or not fibrous tissue, which is considered to be the main cause of adhesion caused by PGA in the pericardial space, could be alleviated by the addition of intermediate water.

The thickest pericardial space observed in Group Y + C at 3 months after the insertion of the PO-OE-coated PGA indicated that the PO-OEs absorbed water and swelled like a hydrogel. It is also suggested that this swelling reduced the inflammatory and foreign body reactions initiated by macrophage migration into the PGA fibers and subsequent fibrotic tissue formation, which may reflect the inhibitory effects on fibroblast adhesion and proliferation on PGA fibers in vitro. Despite these positive expectations, however, the PO-OE coating did not reduce the replacement of PGA with fibrous tissue; thus, there was no obvious difference in the severity of adhesion between the presence and absence of the PO-OE coating.

Taken together, these findings suggest, at least in part, that a polymer used as an antiadhesive should retain intermediate water when hydrated in vivo.

## Limitations

Several limitations associated with the present study warrant mention. First, owing to the small number of experimental animals, it was not possible to statistically prove the anti-adhesive effect of the PO-OE coating. However, we believe this study addresses the hypothesis of positive anti-adhesion effects according to the “intermediate water concept.” Second, the amount of intermediate water added to the PGA fabric was not determined in this study; thus, the amount of intermediate water required to maximize the anti-adhesive effect remains unclear and needs to be defined by evaluating the dose–effect relationship. Third, PO-OE was selected because of its compatibility with the circular knitting machine; however, the half-life, mechanism of absorption, and bonding strength of PO-OE to PGA fibers are unclear. Alternative coating materials and additives can provide intermediate water for biodegradable polymers. Further investigations on the intermediate water concept are necessary for the development of biocompatible and biodegradable polymers as antiadhesive materials.
